# Prognostic factors associated with recurrent COVID-19 and impact of SARS-CoV-2 vaccine on patients with chronic lymphocytic leukemia

**DOI:** 10.3389/fonc.2025.1737446

**Published:** 2026-01-08

**Authors:** Sara Pepe, Roberta Mazzarella, Costanza Andriola, Tania Soriano, Giorgio Sforzini, Francesca Romana Mauro

**Affiliations:** Hematology, Department of Translational and Precision Medicine, Sapienza University of Rome, Rome, Italy

**Keywords:** chronic lymphocytic leukemia, COVID-19, reinfection, SARS-CoV-2, vaccine

## Abstract

**Introduction:**

Patients with chronic lymphocytic leukemia (CLL) are highly vulnerable to infections owing to persistent immune dysfunction, leading to an increased risk of severe disease and reinfection.

**Methods:**

We conducted a retrospective study of 210 CLL patients managed at the Hematology Unit, Sapienza University of Rome, who survived an initial SARS-CoV-2 infection between March 2020 and December 2024. The aim of this study was to assess the incidence, clinical course, and predictors of subsequent COVID-19 events during the Omicron-dominant period.

**Results:**

A subsequent COVID-19 occurred in 71 patients (33.8%), diagnosed after a median of 14 months (IQR, 9–20) from the first episode. Reinfections were significantly more frequent in patients who had experienced their first infection before the Omicron wave (47% vs. 30%; *p* = 0.039). Compared with the initial event, subsequent COVID-19 was milder, with lower rates of pneumonia (8.4% vs. 22.7%; *p* = 0.008), hospitalization (8.4% vs. 25.4%; *p* = 0.002), and no COVID-19–related deaths. Patients on active CLL treatment and those with a Cumulative Illness Rating Scale score ≥6 were at significantly higher risk of reinfection. In multivariate analysis, ongoing CLL therapy (HR 1.92; 95% CI: 1.17–3.16; *p* = 0.010) and elevated comorbidity burden (HR 2.20; 95% CI: 1.30–3.71; *p* = 0.003) independently predicted reinfection. SARS-CoV-2 vaccination did not significantly prevent new infections (36% vs. 33%; *p* = 0.638), but booster doses were associated with prolonged reinfection-free survival (16.3 vs. 9.3 months; *p* = 0.001). These findings indicate that SARS-CoV-2 reinfections remain common but generally mild among CLL patients in the Omicron era. High comorbidity burden and active therapy increase reinfection risk, while booster vaccination may extend protection intervals. Vaccination boosters are essential to reduce COVID-19 morbidity in this immunocompromised population.

## 1 Introduction

In late 2019, during the initial phase of the SARS-CoV-2 outbreak, patients with chronic lymphocytic leukemia (CLL) were identified as highly vulnerable to COVID-19. This susceptibility was largely attributed to their immunocompromised status, which is characterized by impaired humoral and cellular immune responses, rendering them less capable of mounting an effective defense against viral infections (1–6). Consequently, CLL patients experienced high rates of hospitalization and mortality compared to the general population.

With the emergence of the Omicron variants during subsequent pandemic waves, COVID-19 generally manifested with milder clinical outcomes, both in the general population and in immunocompromised individuals (7, 8). A similar trend toward more favorable outcomes was observed among patients with hematologic malignancies, including those with CLL, although their rates of morbidity and mortality continued to exceed those of age-matched individuals without malignancy (9–14). Despite improvements in management and vaccination coverage, patients with CLL remained at elevated risk for severe disease due to persistent immune dysfunction.

On May 5, 2023, the World Health Organization (WHO) officially declared an end to the COVID-19 global emergency status (15). Nevertheless, ongoing circulation of viral variants, along with prolonged viral shedding, has resulted in continued reinfections within the general population during the Omicron wave (16–18). Data on the incidence, severity, and clinical course of subsequent COVID-19 events in patients with CLL during this post-emergency phase remain limited. Consistent with prior observations, older age and a history of prior CLL-directed therapies are anticipated to confer a higher risk of reinfection or severe disease. However, the protective efficacy of SARS-CoV-2 vaccination and the specific risk factors that predispose CLL patients to reinfections have not been fully elucidated.

In this report, we present the findings of a retrospective study designed to evaluate the incidence, clinical characteristics, and outcomes of COVID-19 reinfections in patients with CLL. Our study further investigates the potential impact of prior vaccination and treatment history on susceptibility to subsequent COVID-19 events, aiming to provide evidence-based insights to guide the management of this high-risk population.

## 2 Materials and methods

### 2.1 Patients and COVID-19 diagnosis

This retrospective observational study included 220 patients with CLL who were diagnosed and managed at the Hematology Unit of Sapienza University of Rome in accordance with the International Workshop on Chronic Lymphocytic Leukemia (iwCLL) criteria (19). All patients had experienced COVID-19 between March 2020 and December 2024 (**Supplementary Figure S1**). Of these, 210 patients (95%) survived the initial COVID-19 episode. In line with Italian policy, we recommended to all patients with CLL, including those who had experienced a first COVID-19 event, receiving the annually updated SARS-CoV-2 vaccination, as well as the anti-influenza vaccine. During the follow-up patients were instructed to undergo nasopharyngeal swab testing if they developed symptoms suggestive of COVID-19, including fever, cough, dyspnea, or other typical manifestations.

A subsequent COVID-19 event was defined as a symptomatic SARS-CoV-2 infection occurring more than 90 days after the initial infection, confirmed by a positive nasopharyngeal swab (20). To ensure diagnostic accuracy, all reported swab results were systematically reviewed and verified. We considered COVID-19 to be severe in the presence of at least one of these characteristics: hospitalization, pneumonia, or death due to COVID-19.

The primary objective of this study was to determine the rate of subsequent COVID-19 events among patients with CLL. Secondary objectives included:

1. Characterizing the clinical features, disease course, and survival outcomes of patients who experienced reinfections.
2. Identifying demographic, clinical, and treatment-related factors associated with an increased risk of subsequent infections.
3. Evaluating the protective effect of prior SARS-CoV-2 vaccination on the likelihood of reinfection and clinical severity.

Through this analysis, we aimed to provide a comprehensive assessment of the epidemiology and risk factors for repeated COVID-19 infections in a high-risk hematologic population, thereby informing strategies for prevention and clinical management in CLL patients.

### 2.2 Statistical analysis

We compared the clinical characteristics of patients who experienced a subsequent COVID-19 event to those who did not. Additionally, we analyzed the rates of subsequent COVID-19 events among patients who received the SARS-CoV-2 vaccine after their first COVID-19 event versus those who did not receive the vaccine. We evaluated the prognostic impact of several factors on the risk of subsequent COVID-19 events. These factors included age (a continuous variable), gender (male vs. female), the Cumulative Illness Rating Scale (CIRS) score (≥6 vs. <6), immunoglobulin G levels (≤ 550 mg/dl vs. > 550 mg/dl), TP53 aberration status (present vs. absent), IGHV mutational status (unmutated vs. mutated), treatment naïve status (yes vs. no), CLL treatment after the first COVID-19 event (yes vs. no), and the type of treatment received (venetoclax-based treatment vs. BTK inhibitors treatment). Survival rates were analyzed and compared between patients who developed subsequent COVID-19 and those who did not. We also examined the Reinfection-free survival in patients who experienced a subsequent infection, regardless of whether they had prior SARS-CoV-2 vaccination. To assess differences in patient characteristics, we utilized the chi-square test or Fisher’s exact test for categorical variables, and the Mann–Whitney U-test for continuous variables. Survival probability was evaluated using the Kaplan–Meier method, with group comparisons conducted via the log-rank test. For categorical variables, we employed logistic regression models, while Cox regression models were used for survival analysis in the multivariate analysis. All p-values below 0.05 were considered statistically significant. All analyses were performed using IBM SPSS Statistics, version 27.

## 3 Results

### 3.1 Patients who developed first COVID-19: clinical characteristics

The characteristics of the 210 patients who survived COVID-19 and were included in this study are summarized in **Table 1**. The median age was 68 years. The CIRS score ≥6, and hypogammaglobulinemia (immunoglobulin G levels <550 mg/dL) were present in 16% and 22% of patients, respectively. Unmutated IGHV was recorded in 45% of the cases, and *TP*53 disruption in 23%. Forty-eight (23%) patients were on targeted agents, BTK inhibitors (BTKis), 37 (77%); venetoclax single agent 6 (13%); venetoclax+ anti-CD20 monoclonal antibody, 5 (10%), 30 of whom received treatment as advanced-line therapy.

**Table 1:** Characteristics of patients who developed or did not develop subsequent COVID-19.

	All patients N=210 (%)	Patients with subsequent COVID-19 N=71 (%)	Patients without subsequent COVID-19 N=139 (%)	p value
Median age (IQR)	68 (60-76)	65 (59-75)	69 (60-77)	0.081
Sex, male/female	129/81 (61/39)	44/27(62/38)	85/54 (61/39)	0.907
CIRS ≥6	34 (16)	13 (18)	21 (15)	0.551
IgG levels, ≤ 550 mg/dL	56 (26)	20 (28)	36 (26)	0.724
Beta2-microglobulin, ≥3.5 mg/dL	40 (19)	14 (20)	26 (19)	0.859
*TP*53 disruption	29/125 (23)	10/46 (22)	19/79 (24)	0.767
Unmutated IGHV	73/164 (44.5)	29/57 (51)	44/107 (41)	0.231
CLL treatment after first COVID-19	48 (23)	24 (34)	24 (17)	0.007
No CLL treatment after first COVID-19	162 (77)	47 (66)	115 (83)	
Type of CLL treatment after first COVID-19				
•BTKi	37 (77)	20 (83)†	17 (71)§	0.211
•Venetoclax single agent	6 (13)	1 (4)	5 (21)	
•Venetoclax+ anti-CD20 monoclonal antibody	5 (10)	3 (13)‡	2 (8)¶	
SARS-CoV2 vaccine after the first COVID-19 event	87/210 (41)	31/71 (44)	56/139 (40)	0.568
Median number of vaccine doses after the first COVID-19 event (IQR)	1 (1-2)	1 (1-2)	1 (1-2)	0.610

BTKi, Bruton Tyrosine Kinase inhibitor; CIRS, Cumulative Illness Rating Scale; CLL, chronic lymphocytic leukemia; COVID-19, Coronavirus disease 2019; Ig, immunoglobulins; IGHV, immunoglobulin heavy chain variable region mutations; IQR, interquartile range; *TP*53, tumor protein p53; SARS-CoV-2, severe acute respiratory. syndrome coronavirus 2.

BTKi: †Frontline, 5; advanced line, 15. § Frontline, 11; advanced line, 6.

Venetoclax+ anti-CD20 monoclonal antibody:‡Frontline, 1; advanced line, 2. ¶ Frontline, 1; advanced line, 1.

Despite recommendations during periodic visits, a limited number of patients accepted the invitation to receive the SARS-CoV-2 vaccination, with only 87 out of 210 (41.4%) patients agreed to receive at least one dose of the SARS-CoV-2 vaccine after the first COVID-19 event. After receiving a COVID-19 vaccination, 18 patients (21%) reported mild and transient side effects. These included pain at the injection site in 12 patients, fatigue in 5, and fever in 1.The median follow-up for the 210 patients who survived the first COVID-19 was 31.3 months (95% CI, 30.3-32.6). The median interval between the first COVID-19 event and the SARS-CoV-2 vaccine administration was 6.7 months (IQR, 5.5–9 months), and the median number of vaccine doses patients received was 1 (IQR, 1–2 doses) (**Table 1**).

### 3.2 Patients who developed subsequent COVID-19 event: clinical characteristics

A subsequent COVID-19 event was diagnosed after a median time of 14 months (IQR, 9-20) in 71 out of 210 patients (33.8%). In all patients, the SARS-CoV-2 infection was confirmed through quantitative reverse-transcriptase polymerase chain reaction (RT-qPCR) or a medically supervised rapid antigen test. The 12-month reinfection-free survival rate from the first event was 77.4% (95% CI: 76.8-77.9) (**Supplementary Figure 2**). Additionally, we recorded a third event in 2 patients and a fourth event in 1 patient.

**Figure 1:**
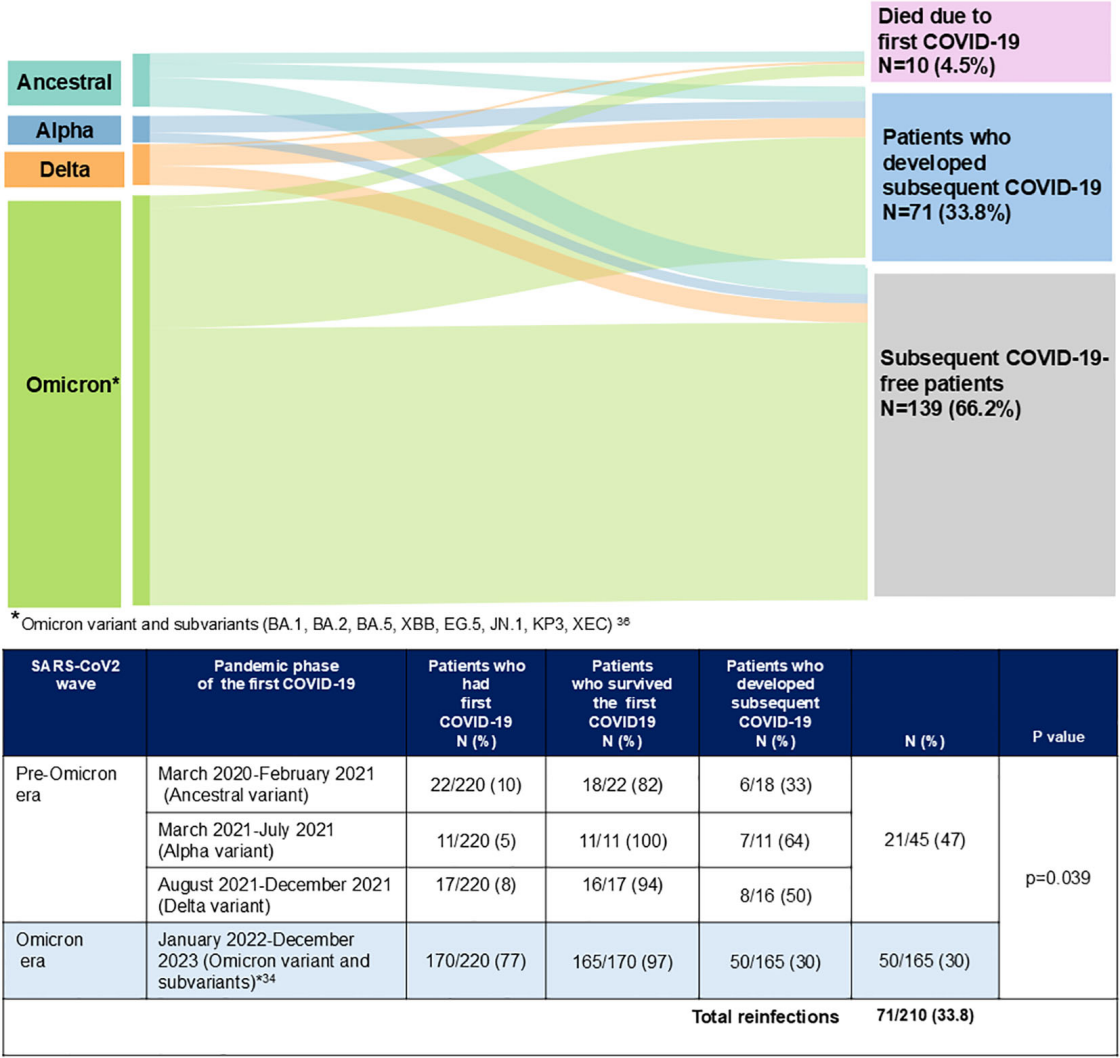
Subsequent COVID-19 events according to the pandemic phase of the first event (21).

As shown in **Figure 1**, all cases of subsequent COVID-19 infections occurred during the Omicron wave. These infections were significantly more frequent in patients who experienced their first event during the pre-Omicron period compared to those who had their initial infection during the Omicron wave (47% vs. 30%, p = 0.039).

#### 3.2.1 Comparison of the characteristics and outcomes of patients who developed first and subsequent COVID-19

**Supplementary Table 1** compares the patient characteristics of patients who developed first and subsequent COVID-19, along with their management and outcomes.

No significant differences in clinical characteristics (age, sex, IgG levels) or CLL genetic features (IGHV and TP53 mutational status, del17p) were observed between patients who developed and did not develop reinfection. Moreover, no significant differences in subsequent COVID-19 rates were observed between patients who received or did not receive SARS-CoV-2 vaccination after the first COVID-19 and in the median number of the vaccines patients had received (**Table 1**). A significantly increased rate of reinfection was observed in patients who had received CLL-directed treatment after the first COVID-19 event (34% vs. 17%; p=0.007), without differences in reinfection rates associated with a specific treatment (BTKi, venetoclax single agent, or venetoclax combined with anti-CD20 monoclonal antibodies).

Overall, the first COVID-19 events were significantly more severe than subsequent events (25.4% vs. 8.4%; p= 0.002). Severe cases were recorded without difference between male and female patients. We have not observed an increase in severe COVID-19 cases related to any treatment, including the combination of rituximab and venetoclax, which was administered to only 5 patients (**Supplementary Table 1**). Compared to first COVID-19 events, reinfections were associated with decreased rates of pneumonia, from 22.7% to 8.4% (p = 0.008), hospitalization, from 25.4% to 8.4% (p = 0.002), need for intensive care, from 4% to 0% (p = 0.119), and fatal cases, from 4.5% to 0% (p = 0.206). Moreover, the duration of CLL-directed treatment interruption was significantly shorter during the second COVID-19 event than the first one [15 days (IQR 14-17) vs. 19 days (IQR 15-25); p = 0.019].

As expected, antiviral agents were used more frequently during the second event than the first one (36.6% vs. 20.9%, p = 0.007). Similarly, the use of monoclonal antibodies increased during the second pandemic event compared to the first one (18.3% vs. 8.2%, p = 0.016).

It is noteworthy that in patients who developed reinfection, ongoing CLL therapy was temporarily suspended, with priority given to antiviral drugs due to their short duration of administration and potential pharmacological interference with targeted agents.

#### 3.2.2 Impact of prior SARS-CoV-2 vaccine on the severity and outcomes of patients who developed subsequent COVID-19

We also compared the severity and outcomes of the 31/87 (36%) patients who developed subsequent COVID-19 after SARS-CoV-2 vaccine and the 40/123 (33%) patients who did not receive the SARS-Cov-2 prior reinfection (**Supplementary Table 1**). The two patient groups did not show significant differences in any of the characteristics analyzed, including age, sex, prior treatments, or types of previous treatments. Additionally, no differences were observed in 24-month OS was observed. The only significant factor that emerged from the comparison of the two groups was that patients who had not previously received the SARS-CoV-2 vaccine experienced a longer interruption in their CLL-directed therapy [16 days (IQR 15-17) vs. 14 days (IQR 14-16); p = 0.023].

### 3.3 Survival and risk factors for subsequent COVID-19 events

Reinfection did not impact survival rates. The 3-year OS rates for patients with and without subsequent COVID-19 were 91.6% and 96.4%, respectively (95% CI: 85.3-97.9 and 91.5-100; p = 0.183).

**Figure 2:**
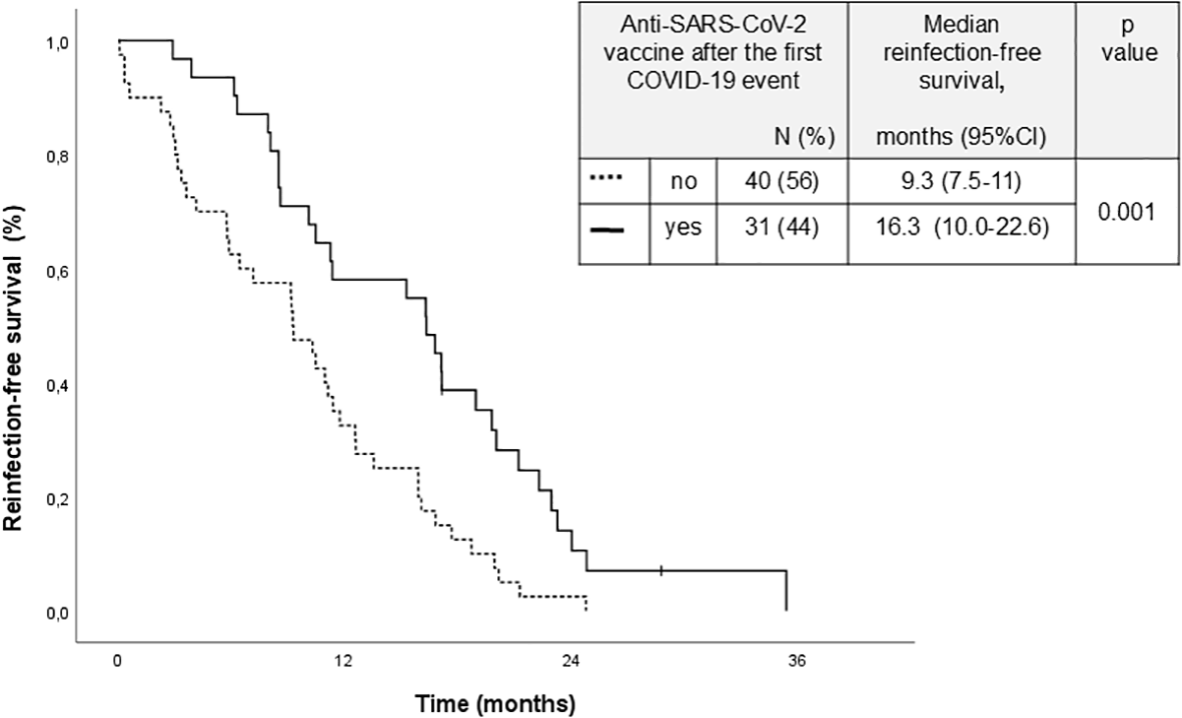
Time to subsequent COVID-19 in patients who received or did not a dose of the anti-SARS-cov-2 vaccine after the first COVID-19 event.

The only two significant and independent factors associated with an increased risk of reinfections were a CIRS score of 6 or higher (HR 2.20, 95% CI: 1.3.-3.71, p = 0.003), and a CLL-directed treatment after the first COVID-19 event CLL (HR 1.92, 95% CI: 1.17-3.16, p = 0.010) (**Supplementary Table 2**). However, no significantly increased risk of reinfection associated with any particular type of treatment was observed. Notably, SARS-CoV2 vaccination did not show a protective impact on the risk of reinfection. However, when we analyzed the group of 71 patients who experienced reinfection we found that those who received at least one booster vaccine after their first infection had significantly longer reinfection-free survival (16.3 months vs. 9.3 months; p = 0.001) (**Figure 2**).

## 4 Discussion

The results of our study indicate that subsequent COVID-19 events are not uncommon in patients with CLL, with a prevalence of 33.8%. However, it should be noted that the reinfection rate we found may be underestimated, as mild symptoms may result in the omission of nasopharyngeal swabs, leading to missed diagnoses. Subsequent COVID-19 events were more frequent among patients who experienced their first event during the pre-Omicron period than during the Omicron wave period. This finding may be attributed not only to reduced immunological protection caused by the emergence of virus variants but also to the shorter study follow-up duration for patients who developed COVID-19 during the Omicron period.

Interestingly, a Chinese prospective registry that included patients with hematologic disorders reported a similar rate of subsequent COVID-19 events at 31.5% (22). In contrast, a study from the European Research Initiative on CLL (ERIC) group reported a lower reinfection rate of 6.65%. This discrepancy may be attributed to the shorter observation period in that study (23).

Reinfections appear to be more frequent in older populations, such as those diagnosed with CLL, compared to the general population. A recent systematic review and meta-analysis encompassing data from 28 studies revealed that the cumulative incidence of reinfections during the Omicron period was 3.35%, with a higher rate of 6.62% observed in adults aged 18–59 years (18). In this study, ongoing treatment and a high CIRS were two significant and independent risk factors for subsequent COVID-19 infection. It is important to emphasize that these same factors appeared associated with an increased risk for a more severe outcome of COVID-19 in patients with CLL during initial phases (23).

Given the continuous risk of reinfections due to mutant viruses, a preventive policy for SARS-CoV-2 infections with annually updated vaccinations has been recommended in European countries, and the United States in older and immunocompromised patients. This recommendation remains relevant as an increase in the SARS-CoV-2 activity with the emergence of new Omicron-descendant variants and a rise in infections in some countries worldwide have been reported by the WHO (24) and the European Centre for Disease Prevention and Control (ECDC) (25). However, as we observed in patients included in this study there has been a progressive decline in the number of vaccinated individuals in recent years, resulting in suboptimal coverage in COVID-19 vaccination (26–28).

The decrease in interest in vaccines can be attributed to many factors. The immunity already achieved through prior infections and vaccinations, the reduced virulence of mutated viral forms, and the availability of effective antiviral drugs and monoclonal antibodies have made reinfections less severe than those diagnosed during the initial outbreaks. Moreover, the spread of mutant variants has diminished the effectiveness of vaccines in preventing infections and has discouraged vaccinations, as well as the low antibody responses in observed in patients with CLL (4). However, there are solid data suggesting that booster vaccines effectively promote immune responses, even in patients with CLL or other hematologic malignancies who have treatment-related severe immunodeficiency (5, 6, 29–32). Recent data from a retrospective cohort study involving 72,831 individuals with cancer supports the favorable impact of vaccine boosters, resulting in a reduced rate of hospitalization and ICU admissions (33). Notably, recent data from a randomized study by Cook et al. (34) demonstrated that it is not necessary to pause BTKi monotherapy in patients with CLL around the time of SARS-CoV-2 vaccination to improve a specific antibody response. This finding has an important practical impact as eliciting effective immune responses without the need to discontinue or delay treatment may help extend the use of booster vaccines in patients with CLL. Data from our study indicate that while repeated immunization with booster SARS-CoV-2 vaccine did not prevent subsequent COVID-19, it could delay the onset of the next infections. This finding suggest that boosting immune memory may effectively extend the infection-free period in patients receiving targeted agents. This is not a negligible benefit for many CLL patients receiving continuous therapy.

Previous studies showed low severity of COVID-19 during the Omicron variant’s spread, even in CLL patients (7–11, 17, 18). In this study, all reinfections diagnosed during the Omicron wave were also of low severity, with no fatal cases. Antiviral therapy also played an important role in reducing the severity of subsequent COVID-19. It should be noted that in this study, we suspended CLL-directed therapy in patients with reinfection and prioritized antiviral therapy given its short duration. However, it is important to note that 6 older patients included in this study exhibited less favorable outcomes, with prolonged pneumonia and respiratory distress.

We recognize the limitations of this study, including its retrospective design, the relatively small sample size of patients, and the possibility of missed cases of reinfection. Additionally, we did not have access to viral genome sequencing data for the variants involved in reinfection, nor did we have information on humoral and cellular immune responses.

Despite these limitations, this real-world study suggests that during the Omicron wave, SARS-CoV-2 reinfections were relatively common among patients with CLL, especially in those with a high burden of comorbidities and those undergoing active treatment. Immunocompromised patients should be aware of the ongoing risk of SARS-CoV-2 infection/reinfection. Targeted booster strategies are crucial for mitigating COVID-19 in patients with impaired immunity. Our findings highlight the advantages of receiving SARS-CoV-2 vaccine boosters, which help delay the onset of subsequent COVID-19 and reduce the duration of CLL treatment interruptions during reinfection. This data support the recommendation from the CLL guidelines, which advocate for vaccinations in all patients with CLL (35, 36).

Adequate information is also essential as it enables early diagnosis and treatment. Additionally, targeted social and behavioral measures, such as masking in indoor public settings with high transmission and maintaining physical distancing, have been proven to significantly reduce viral exposure and provide extra protection against SARS-CoV-2 infection.

## Data Availability

The raw data supporting the conclusions of this article will be made available by the authors, without undue reservation.

## References

[1] Scarfò L Chatzikonstantinou T Rigolin GM Quaresmini G Motta M Vitale C . COVID-19 severity and mortality in patients with chronic lymphocytic leukemia: a joint study by ERIC, the European Research Initiative on CLL, and CLL Campus. Leukemia. (2020) 34:2354–63. doi: 10.1038/s41375-020-0959-x, PMID: 32647324 PMC7347048

[2] Mato AR Roeker LE Lamanna N Allan JN Leslie L Pagel JM . Outcomes of COVID-19 in patients with CLL: a multicenter international experience. Blood. (2020) 136:1134–43. doi: 10.1182/blood.2020006965, PMID: 32688395 PMC7472711

[3] Chatzikonstantinou T Kapetanakis A Scarfò L Karakatsoulis G Allsup D Cabrero AA . COVID-19 severity and mortality in patients with CLL: an update of the international ERIC and Campus CLL study. Leukemia. (2021) 35:3444–54. doi: 10.1038/s41375-021-01450-8, PMID: 34725454 PMC8559135

[4] Roeker LE Knorr DA Pessin MS Ramanathan LV Thompson MC Leslie LA . Anti-SARS-CoV-2 antibody response in patients with chronic lymphocytic leukemia. Leukemia. (2020) 34:3047–9. doi: 10.1038/s41375-020-01030-2, PMID: 32855439 PMC7450257

[5] Herishanu Y Avivi I Aharon A Shefer G Levi S Bronstein Y . Efficacy of the BNT162b2 mRNA COVID-19 vaccine in patients with chronic lymphocytic leukemia. Blood. (2021) 137:3165–73. doi: 10.1182/blood.2021011568, PMID: 33861303 PMC8061088

[6] Mauro FR Giannarelli D Galluzzo CM Visentin A Frustaci AM Sportoletti P . Antibody response to the SARS-coV-2 vaccine and COVID-19 vulnerability during the omicron pandemic in patients with CLL: two-year follow-up of a multicenter study. Cancers (Basel). (2023) 15:2993. doi: 10.3390/cancers15112993, PMID: 37296954 PMC10251854

[7] Kohn M Alsuliman T Lamure S Cheminant M Delage J Merle De Boever C . Characteristics of SARS-CoV-2 infection in lymphoma/chronic lymphocytic leukemia patients during the Omicron outbreak. Leuk Lymphoma. (2022) 63(11):2686–90. doi: 10.1080/10428194.2022.2086249, PMID: 35719107

[8] Relan P Motaze NV Kothari K Askie L Le Polain O Van Kerkhove MD . Severity and outcomes of Omicron variant of SARS-CoV-2 compared to Delta variant and severity of Omicron sublineages: a systematic review and metanalysis. BMJ Glob Health. (2023) 8:e012328. doi: 10.1136/bmjgh-2023-012328, PMID: 37419502 PMC10347449

[9] World Health Organization . Severity of disease associated with omicron variant as compared with delta variant in hospitalized patients with suspected or confirmed SARS-coV-2 infection. Available online at: https://iris.who.int/bitstream/handle/10665/354794/9789240051829-eng.pdf?sequence=1 (Accessed August, 13, 2025).

[10] Pagano L Salmanton-García J Marchesi F Busca A Corradini P Hoenigl M . COVID-19 infection in adult patients with hematological Malignancies: a European Hematology Association Survey (EPICOVIDEHA). J Hematol Oncol. (2021) 14:168. doi: 10.1186/s13045-021-01177-0, PMID: 34649563 PMC8515781

[11] Bronstein Y Gat R Levi S Cohen YC Luttwak E Benyamini N . COVID-19 in patients with lymphoproliferative diseases during the Omicron variant surge. Cancer Cell. (2022) 40:578–80. doi: 10.1016/j.ccell.2022.04.015, PMID: 35477028 PMC9021039

[12] Niemann CU da Cunha-Bang C Helleberg M Ostrowski SR Brieghel C . Patients with CLL have a lower risk of death from COVID-19 in the Omicron era. Blood. (2022) 140:445–50. doi: 10.1182/blood.2022016147, PMID: 35588468 PMC9122776

[13] Salmanton-García J Marchesi F Farina F Weinbergerová B Itri F Dávila-Valls J . Decoding the historical tale: COVID-19 impact on haematological Malignancy patients-EPICOVIDEHA insights from 2020 to 2022. EClinicalMedicine. (2024) 71:102553. doi: 10.1016/j.eclinm.2024, PMID: 38533127 PMC10963230

[14] Martínez JC Sica RA Stockerl-Goldstein K Rubinstein SM . COVID-19 in patients with hematologic Malignancies: outcomes and options for treatments. Acta Haematol. (2022) 145:244–56. doi: 10.1159/000522436, PMID: 35134811 PMC9059013

[15] Statement on the fifteenth meeting of the IHR . Emergency Committee on the COVID-19 pandemic (2005). Available online at: https://www.who.int/news/item/05-05-2023-statement-on-the-fifteenth-meeting-of-the-international-health-regulations-(2005)-emergency-committee-regarding-the-coronavirus-disease-(covid-19)-pandemic (Accessed August, 13, 2025).

[16] Chemaitelly H Ayoub HH Tang P Hasan MR Coyle P Yassine HM . Immune imprinting and protection against repeat reinfection with SARS-coV-2. N Engl J Med. (2022) 387:1716–8. doi: 10.1056/NEJMc2211055, PMID: 36223534 PMC9634858

[17] Abuhasira R Burrack N Nesher L Ostrovsky D Novack V . Characteristics and clinical outcomes of patients with COVID-19 recurrent infection during the omicron variant predominance. Eur J Intern Med. (2023) 114:131–4. doi: 10.1016/j.ejim.2023.05.017, PMID: 37198012 PMC10183632

[18] Kulkarni D Lee B Ismail NF Rahman AE Spinardi J Kyaw MH . Incidence, severity, risk factors and outcomes of SARS-CoV-2 reinfections during the Omicron period: a systematic review and meta-analysis. J Glob Health. (2025) 15:4032. doi: 10.7189/jogh.15.04032, PMID: 39916552 PMC11803431

[19] Hallek M Cheson BD Catovsky D Caligaris-Cappio F Dighiero G Döhner H . iwCLL guidelines for diagnosis, indications for treatment, response assessment, and supportive management of CLL. Blood. (2018) 131:2745–60. doi: 10.1182/blood-2017-09-806398, PMID: 29540348

[20] Available online at: https://www.epicentro.iss.it/coronavirus/bollettino/Bollettino-sorveglianza-integrata-COVID-19_18-gennaio-2023.pdf (Accessed August, 13, 2025).

[21] Available online at: https://www.epicentro.iss.it/coronavirus/bollettino/Bollettino-sorveglianza-integrata-COVID-19_29-gennaio-2025.pdf (Accessed August, 13, 2025).

[22] Li J Zhang Y Liu Z Yang Z Liu L Xu G . Increased SARS-CoV-2 reinfection frequency, attenuated severity, and risk factor analysis in patients with hematological Malignancies. J Infect. (2024) 89:106233. doi: 10.1016/j.jinf.2024.106233, PMID: 39067795

[23] Visentin A Chatzikonstantinou T Scarfò L Kapetanakis A Demosthenous C Karakatsoulis G . The evolving landscape of COVID-19 and post-COVID condition in patients with chronic lymphocytic leukemia: A study by ERIC, the European research initiative on CLL. Am J Hematol. (2023) 98:1856–68. doi: 10.1002/ajh.27093, PMID: 37772428

[24] Available online at: https://data.who.int/dashboards/covid19/summary (Accessed August, 13, 2025).

[25] Available online at: https://www.ecdc.europa.eu/en/news-events/slow-increases-covid-19-observed-across-eueea-new-variant-emerges (Accessed August, 13, 2025).

[26] Available online at: https://www.cdc.gov/covidvaxview/weekly-dashboard/adults-65yrs-older-vaccination.html (Accessed August, 13, 2025).

[27] Available online at: https://www.cdc.gov/covidvaxview/weekly-dashboard/index.html (Accessed August, 13, 2025).

[28] Available online at: https://www.ecdc.europa.eu/en/publications-data/covid-19-vaccination-coverage-eueea-during-2024-25-season-campaigns (Accessed August, 13, 2025).

[29] Benjamini O Gershon R Bar-Haim E Lustig Y Cohen H Doolman R . Cellular and humoral response to the fourth BNT162b2 mRNA COVID-19 vaccine dose in patients with CLL. Eur J Haematol. (2023) 110:99–108. doi: 10.1111/ejh.13878, PMID: 36208015 PMC9874468

[30] Campanella A Capasso A Heltai S Taccetti C Albi E Herishanu Y . Additional booster doses in patients with chronic lymphocytic leukemia induce humoral and cellular immune responses to SARS-CoV-2 similar to natural infection regardless ongoing treatments: A study by ERIC, the European Research Initiative on CLL. Am J Hematol. (2024) 99:745–50. doi: 10.1002/ajh.27218, PMID: 38264829

[31] Abid MB Rubin M Szabo A Longo W Fenske TS McCoy C . Efficacy of multiple SARS-coV-2 vaccine doses in patients with B cell hematologic Malignancies receiving chimeric antigen receptor T cell therapy: A contemporary cohort analysis. Transplant Cell Ther. (2024) 30:285–97. doi: 10.1016/j.jtct.2023.12.011, PMID: 38142942

[32] Herishanu Y Rahav G Levi S Braester A Itchaki G Bairey O . Efficacy of a third BNT162b2 mRNA COVID-19 vaccine dose in patients with CLL who failed standard 2-dose vaccination. Blood. (2022) 139:678–85. doi: 10.1182/blood.2021014085, PMID: 34861036 PMC8648353

[33] Skarbinski J Elkin EP Ziemba YC Kazemian E Wilson BM Siddiqui H . COVID-19 vaccine booster uptake and effectiveness among US adults with cancer. JAMA Oncol. (2025) 2025:e252020. doi: 10.1001/jamaoncol.2025.2020, PMID: 40674059 PMC12272354

[34] Cook JA Patten PEM Peckham N Moss P Phillips N Abhishek A . A 3-week pause versus continued Bruton tyrosine kinase inhibitor use during COVID-19 vaccination in individuals with chronic lymphocytic leukaemia (IMPROVE trial): a randomised, open-label, superiority trial. Lancet Haematol. (2025) 12:e294–303. doi: 10.1016/S2352-3026(25)00008-0, PMID: 40175001

[35] National Comprehensive Cancer Network . NCCN Clinical Practice Guidelines in Oncology: Chronic Lymphocytic Leukemia/Small Lymphocytic Lymphoma. Version 1.2026. Fort Washington, PA: National Comprehensive Cancer Network. (2025). Available online at: https://www.nccn.org/professionals (Accessed December 21, 2025).

[36] Walewska R Eyre TA Bloor A Follows G Iyengar S Johnston R . 2025 British Society for Haematology Guideline for the treatment of chronic lymphocytic leukaemia. Br J Haematol. (2025) 00:1–18. doi: 10.1111/bjh.70100, PMID: 41069109

