# Supplementary material for: Prognostic factors associated with recurrent COVID-19 and impact of SARS-CoV-2 vaccine on patients with chronic lymphocytic leukemia
Source: Front Oncol. 2026 Jan 8;15:1737446. doi: 10.3389/fonc.2025.1737446 (PMC12823520; doi:10.3389/fonc.2025.1737446)
Supplement: Supplementary file 1 [file Supplementaryfile1.pdf]

SUPPLEMENTARY INFORMATION

1. Supplementary Figure 1. Patient disposition and analyses made on study data.

2. Supplementary Figure 2. Reinfection-free Survival

3. Supplementary Table.1. Clinical characteristics and outcomes of patients at the first and subsequent COVID-19 event.

4. Supplementary Table 2. Reinfection-free Survival: Univariate and multivariate analysis

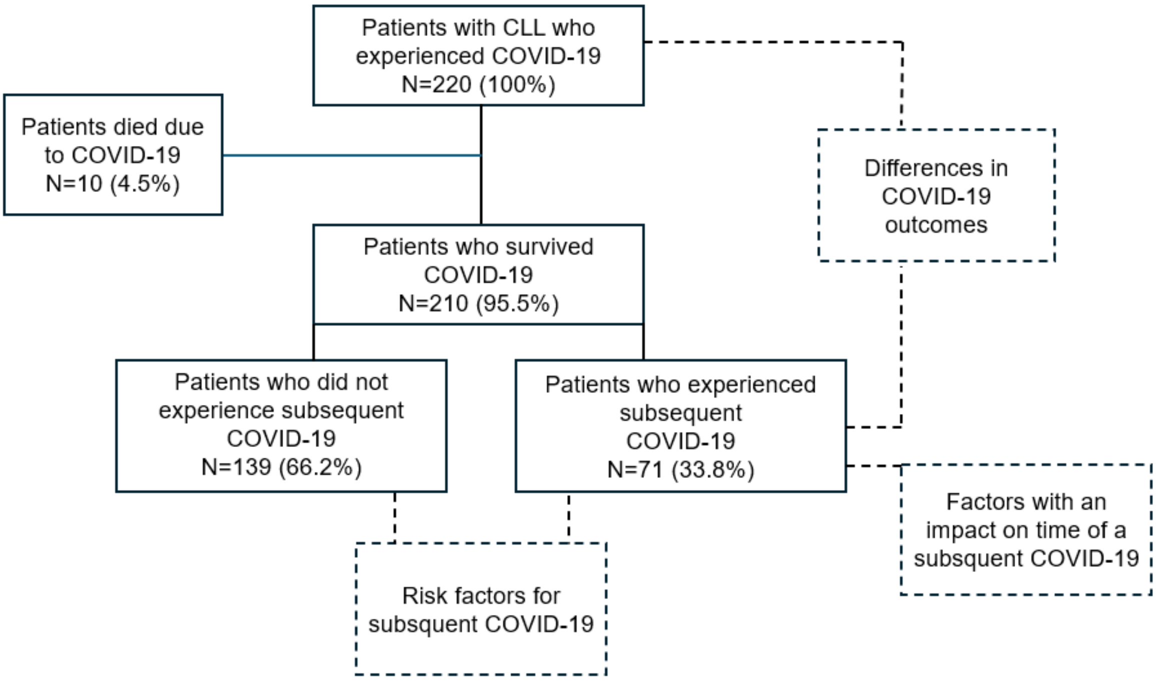

Supplementary Fig. S1. Patients disposition and analyses made on study data.

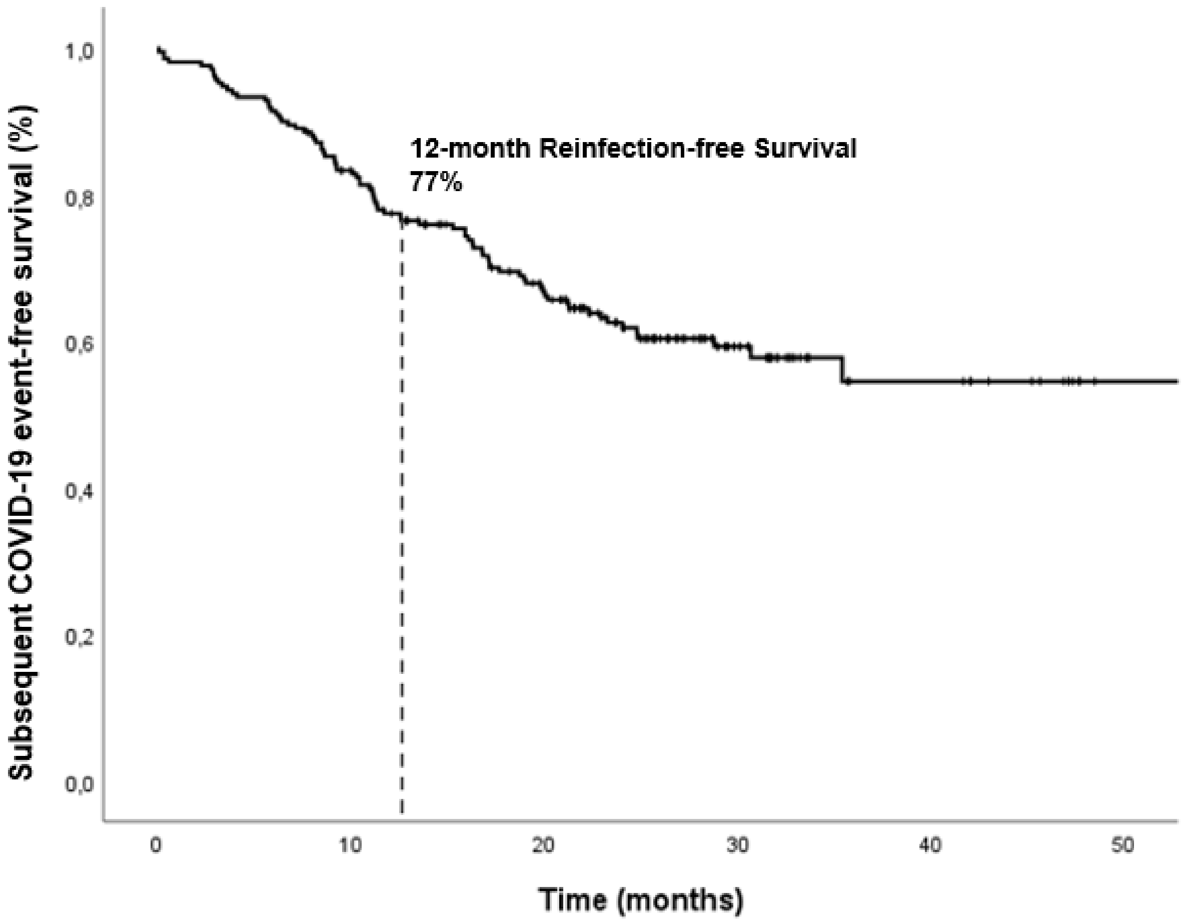

Supplementary Fig.S2. Reinfection-free Survival.

Supplementary Tab. S1. Clinical characteristics and outcomes of patients at the first and subsequent COVID-19 event.

	First COVID-19 N=220 (%)	Subsequent COVID-19 N=71 (%)	p value	Characteristics and outcomes of patients at subsequent COVID-19 according to the SARS-CoV-2 vaccination		
				Patients who received the vaccine[(1)]	Patients who did not receive the vaccine	p value
No patients	N=220 (%)	N=71 (%)	-	N=31 (%)	N=40 (%)	-
Median age, years	69 (60-77)	65 (59-75)	0.589	67 (59-75)	68 (61-78)	0.412
Sex; male/female	139/81 (63/37)	44/27 (62/38)	0.888	18/13 (58/42)	26/14 (65/35)	0.550
CLL Treatment at the time of COVID-19	64 (29.1)	29 (40.8)	0.080	14 (45)	10 (25)	0.084
Severe COVID-19	56 (25.4)	6 (8.4)	0.002	2 (6)	4 (10)	0.689
• Pneumonia	50 (22.7)	6 (8.4)	0.008	2 (6)	4 (10)	0.689
• Hospitalized [Intensive care unit %]	56 (25.4) [4]	6 (8.4) [0]	0.002	2 (6)	4 (10)	0.689
• COVID-19-related deaths	10 (4.5)	0 (0)	0.126	0	0	-
Severe COVID-19 in male/female patients	34/22 (61/39)	3/3 (50/50)	0.611	0/2 (0/100)	3/1 (75/25)	0.400
Previous treatment directed to CLL in patients with severe COVID-19 [(2)]<br>• BTKi- based treatment<br>• Venetoclax + anti-CD20 monoclonal antibody	22/56 (39.3)<br>2/56 (3.6)	2/6 (33.3)<br>1/6 (16.7)	0.745	0<br>0	2/4 (50)<br>1/4 (25)	1
No treatment directed to CLL in patients with severe COVID-19	32/56 (57.1)	3/6 (50)	0.922	2/2 (100)	1/4 (25)	0.400-
Median duration of interruption of CLL treatment due to COVID-19, days (IQR)	19 (15-25)	15 (14-17)	0.019	14 (14-16)	16 (15-17)	**0.023**
Treatment for COVID-19 [(3)]	64 (29)	39 (55)	0.001	18 (58)	21 (53)	0.820
• Antiviral agents [(4)]	46 (20.9)	26 (36.6)	0.007	12 (39)	14 (35)	0.806
• Anti-Sars-CoV-2 monoclonal antibodies [(5)]	18 (8.2)	13 (18.3)	0.016	6 (19)	7 (18)	1
% 24-month OS	91	96	0.164	100	91.4	0.117

Abbreviations: CLL, chronic lymphocytic leukemia; COVID-19, Coronavirus disease 2019; IQR, interquartile range; OS, Overall Survival.

[(1)] Types of the anti-SARS-CoV2 vaccine administered in all vaccinated patients after the first COVID-19. event: BNT162b2 mRNA, 85 (98%) patients; mRNA-1273, 2 (2%) patients; in patients who experienced reinfection: BNT162b2 mRNA, 30 (97%) patients; mRNA-1273, 1 (3%) patient.

[(2)] All patients who had previously received chemoimmunotherapy had discontinued anti-CD20 monoclonal antibody treatment more than 24 months before the COVID-19 event.

[(3)] 64 (29%) patients received treatment for the first COVID19 event and 39 (55%) for the subsequent COVID-19 event

[(4)] Antiviral agents used during the first COVID-19 episode: remdesivir, 21 (46%) patients; nirmatrelvir/ritonavir, 25 (54%) patients. Antiviral agents used during the second COVID-19 episode: remdesivir, 6 (23%) patients; nirmatrelvir/ritonavir, 20 (77%) patients.

[(5)] Anti-SARS-CoV-2 monoclonal antibodies: sotrovimab in 10 (56%) patients and casirivimab/imdevimab in 8 (44%) patients at the first COVID-19; sotrovimab in 10 (77%) patients and casirivimab/imdevimab in 3 (23%) patients at reinfection**.**

Supplementary Tab.S2. Risk of subsequent COVID-19: univariable and multivariable analysis,

	Univariable analysis		Multivariable analysis	
	HR (95%CI)	p value	HR (95%CI)	p value
Age, years	1.00 (0.99-1.03)	0.521	-	-
Sex<br>male vs. female	0.99 (0.63-1.56)	0.976	-	-
CIRS<br>≥6 vs. <6	2.73 (1.66-4.47)	<0.001	2.20 (1.30-3.71)	0.003
IgG levels, mg/dl<br>≤ 550 vs. >550	1.31 (0.81-2.12)	0.278	-	-
*TP*53 aberration<br>present vs. absent	1.55 (0.84-2.87)	0.165	-	-
IGHV mutational status<br>unmutated vs. mutated	1.52 (0.93-2.48)	0.096	-	-
CLL treatment after the first COVID-19 event<br>yes vs. no	2.38 (1.50-3.79)	<0.001	1.92 (1.17-3.16)	0.010
Number of prior treatments<br>≥2 vs. 1	1.62 (0.90-2.96)	0.110	-	-
Treatment duration<br>fixed vs continuous treatment	1.33 (0.54-3.26)	0.541	-	-
Type of continuous therapy<br>venetoclax vs. BTKi	0.46 (0.12-1.96)	0.292	-	-
Venetoclax-based therapy<br>Venetoclax single agent vs.<br>Venetoclax+ anti-CD20 monoclonal antibody	0.75 (0.13-4.54)	0.758	-	-
SARS-CoV-2 vaccine after first COVID-19<br>yes vs. no	0.83 (0.53-1.30)	0.409	-	-

Abbreviations: BTKi, Bruton Tyrosine Kinase inhibitor; CIRS, Cumulative Illness Rating Scale; CLL, chronic lymphocytic leukemia; COVID-19, Coronavirus disease 2019; FD, fixed-duration; Ig, immunoglobulins; IGHV, immunoglobulin heavy chain variable region mutations; HR, hazard ratio; IQR, interquartile range; TP53, tumor protein p53; SARS-CoV-2, severe acute respiratory syndrome coronavirus 2.
